# *Wolbachia* in Black Spiny Whiteflies and Their New Parasitoid Wasp in Japan: Evidence of the Distinct Infection Status on *Aleurocanthus camelliae* Cryptic Species Complex

**DOI:** 10.3390/insects13090788

**Published:** 2022-08-31

**Authors:** Eko Andrianto, Atsushi Kasai

**Affiliations:** 1 Science of Biological Environment, The United Graduate School of Agricultural Science (UGSAS), Gifu University, Gifu City 501-1193, Japan; 2 Department of Bioresource Sciences, Faculty of Agriculture, Shizuoka University, Shizuoka City 422-8528, Japan

**Keywords:** *Aleurocanthus* cf. *A. spiniferus*, *Eretmocerus* sp. recombination, oscillation hypothesis, *wAlec*

## Abstract

**Simple Summary:**

The *Aleurocanthus camelliae* cryptic species complex, which includes a number of morphospecies and/or haplotypes, is one of the growing biological issues, the underlying mechanism of which is still unknown. It is well-known that *Wolbachia* infection can produce significant mitochondrial divergence in insects, which may eventually result in cryptic speciation. Therefore, the diversity and phenotypic characteristics of *Wolbachia* natural infections in the *A. camelliae* cryptic species complex were investigated. Two morphospecies were found to have distinct infection statuses. *A. spiniferus* morphospecies was the uninfected population, while *A. camelliae* morphospecies was fixed for infections. The oscillation hypothesis is discussed in light of the current discovery of novel cryptic species of *A. camelliae*. This idea may offer insights into cryptic speciation, specifically on how specialization and host expansion have been observed among these species. Additionally, this research discovered a parasitoid wasp from the genus *Eretmocerus* in *A. camelliae* for the first time in Japan.

**Abstract:**

*Wolbachia*, an alphaproteobacterial reproductive parasite, can cause profound mitochondrial divergence in insects, which might eventually be a part of cryptic speciation. *Aleurocanthus camelliae* is a cryptic species complex consisting of several morphospecies and/or haplotypes that are genetically different but morphologically indistinctive. However, little is known about the *Wolbachia* infection status in these tea and *Citrus* pests. Thus, this study aimed to profile the diversity and phenotypic characteristics of *Wolbachia* natural infections in the *A. camelliae* cryptic species complex. A monophyletic strain of *Wolbachia* that infected the *A. camelliae* cryptic species complex (*wAlec*) with different patterns was discovered. Whiteflies that are morphologically identical to *Aleurocanthus spiniferus* (*Aleurocanthus* cf. *A. spiniferus* in *Eurya japonica* and *A. spiniferus* in *Citrus*) were grouped into uninfected populations, whereas the fixed infection was detected in *A. camelliae* B1 from Theaceae. The rapid evolution of *wAlec* was also found to occur through a high recombination event, which produced subgroups A and B in *wAlec*. It may also be associated with the non-cytoplasmic incompatibility (CI) phenotype of *wAlec* due to undetectable CI-related genes from phage WO (*WOAlec*). The current discovery of a novel cryptic species of *A. camelliae* led to a discussion about the oscillation hypothesis, which may provide insights on cryptic speciation, particularly on how specialization and host expansion have been recorded among these species. This study also identified a parasitoid wasp belonging to the genus *Eretmocerus* in *A. camelliae*, for the first time in Japan.

## 1. Introduction

*Wolbachia* is a well-known reproductive parasite that is one of the most common facultative symbiotic bacteria (secondary symbionts) of insects [1,2] and a speciation agent [3]. *Wolbachia* has a wide range of relationships with the host, from facultative parasitic to obligate mutualist [4]. Fixed infections (obligate mutualist) and phenotypic strain diversity (facultative parasitic) are important characteristics of *Wolbachia* infections associated with their significant roles in the induction of parthenogenesis and cytoplasmic incompatibility (CI), respectively [3]. *Wolbachia*, in its more extreme role as a speciation agent, *Wolbachia* may reduce gene flow between geographically distant and genetically distinct populations that overlap before the reproductive barrier mechanisms are complete [5]. Cryptic species complex, a group of genetically different but morphologically indistinctive species, is an emerging biological problem also observed in whiteflies (Hemiptera: Aleyrodidae). Increasing reports suggest the effects of *Wolbachia* infection on the mitochondrial diversity and evolution of hosts, supporting the hypothesis that cryptic speciation is related to *Wolbachia* infections [6,7,8,9,10].

High vigilance must be given to the increasing facts about intercepted whiteflies at the plant quarantine that might also be invasive species, as they would have major environmental and economic consequences. A case in point is the interception of the whitefly *Aleurocanthus spiniferus* in Japan. This species was first found in 1919 in Kagoshima Prefecture. Due to a lack of natural enemies, it subsequently became a serious pest in citrus orchards on Kyushu Island, Japan [11,12]. Interestingly, some secondary symbionts are supportive agents for whitefly cryptic species complex invasion, such as the sweet potato whitefly *Bemisia tabaci* [13]. They confer adaptive responses that eventually support the invasion of this pest. For example, *Wolbachia* promotes fitness and provides some protection against the parasitism of parasitoid wasps [14]. However, it is yet to be determined just how common these phenotypic effects are to be found in other whiteflies.

The Camellia spiny whitefly *Aleurocanthus camelliae* (Hemiptera: Aleyrodidae) cryptic species complex is a pest to the Theaceae plants that originated from China and is currently considered to be an invasive species, as it has been detected in Japan (2004), the Netherlands (2018), Italy (2020), and Indonesia (2020) [15,16,17,18,19]. The *A. camelliae* cryptic species complex consists of at least three related species (*Aleurocanthus woglumi*, *Aleurocanthus spiniferus*, and *A. camelliae*) [16] and five associated haplotypes (*A. camelliae* haplotypes B1–B3 and *A. spiniferus* haplogroup A1 and A2) [19,20]. *A. spiniferus* is extremely polyphagous [21]. Conversely, *A. camelliae* prefers mostly Theaceae plants and is not inhabit *Citrus* plants (Rutaceae) as their host [22], although they could also be found in *Zanthoxylum piperitum* (Rutaceae) [23]. Thus, their dispersion was strongly associated with Theaceae mobility through human activities, such as the global trading of Theaceae plants, such as *Camellia sinensis*, *Camellia japonica*, *Camellia sasanqua*, and *Eurya japonica*. However, the association between *A. camelliae* cryptic species complex and bacterial symbionts is poorly understood. There are limited studies related to this topic and other close species that have been examined, such as *A. woglumi* [24] and *A. spiniferus* [25].

Therefore, this study aimed to examine the infection status and diversity of *Wolbachia* in the *A. camelliae* cryptic species complex in Japan, including *A. camelliae* haplotype B1, *A. spiniferus* haplogroup A1, and a novel cryptic species complex (*Aleurocanthus* cf. *A. spiniferus*). In addition, to detect the possibility of the horizontal transfer mechanism of Wolbachia, the associated population of insects such as *Pealius euryae*, another Theaceae whitefly that was newly found to inhabit *C. sinensis* in the fields (Shizuoka and Kyoto Prefectures) and parasitoid wasps. The infection and diversity of *Wolbachia* in *A. camelliae* cryptic species complex were determined using single-gene typing and multilocus sequence typing (MLST). Moreover, its phenotypic characteristics were examined via molecular detection of CI-related genes.

## 2. Materials and Methods

### 2.1. Sample Collection

**Figure 1:**
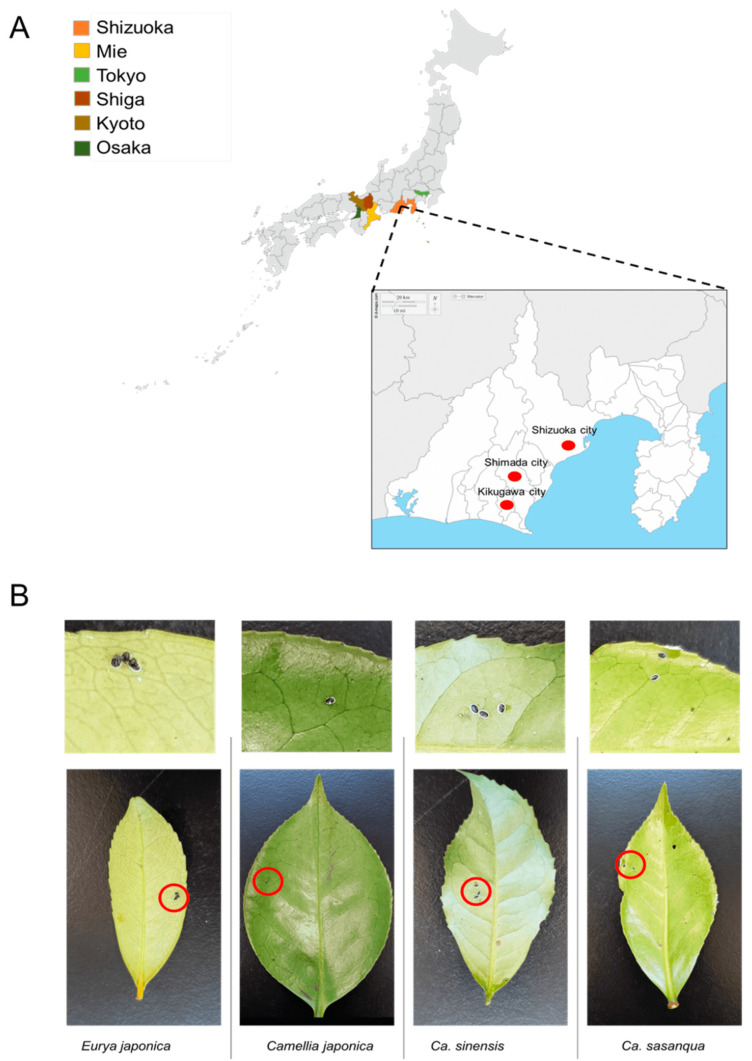
Sample collection: (**A**) sampling sites; (**B**) representative samples of Camellia spiny whitefly and *A. camelliae* nymphs for molecular assessment.

From 2017 to 2022, samples were collected in six Prefectures in Japan from tea (*C. sinensis*) fields and Theaceae plants, including *E. japonica*, *C. sasanqua*, and *C. japonica*. Samples included in the sample collection stocks were collected between 2009 and 2011 from the Laboratory of Applied Entomology, Shizuoka University [26]. The survey was conducted in Shizuoka Prefecture, Shizuoka City, Shimada City, and Kikugawa City. Other prefectures, such as Osaka, Kyoto, Tokyo, Shiga, and Mie, were also evaluated (Figure 1A). From March 2021 to February 2022, systematic random sampling was employed in a tea field in which many tea varieties (*C. sinensis*) grow to estimate the dynamics of the positivity rate of *Wolbachia* infection in the field. This field belongs to the National Agriculture and Food Research Station in Kanaya–Shimada, Shizuoka Prefecture. The leaves infested by a small number of whiteflies were selected as representative samples (Figure 1B; Table 1) and assumed to be a single colony of individuals from different parents. The specimens were stored in a freezer at −20 °C for future deoxyribonucleic acid DNA extraction. 

### 2.2. DNA Extraction 

The DNA of *Wolbachia* and its hosts was extracted using a slightly modified HotShot method [27] in two steps using Alkaline Buffer (25 mM NaOH and 0.2 EDTA) and a neutralizing solution (40 mM Tris-HCl pH 5.5). Using power masher II for Biomasher II, one individual nymph of whiteflies was crushed in an Eppendorf tube containing 50 μL of Alkaline Buffer. Therefore, aliquots of ~30 μL were transferred into 200 μL tubes and placed in a thermocycler at 95 °C for 15 min. The temperature was reduced to 4 °C, and 30 μL of the neutralizing solution was added and vortexed for 10 s.

### 2.3. Morphomolecular Identification

Morphological identification was performed using keys on species of the genus *Aleurocanthus* [28] to determine the species. Morphological comparison between *A. spiniferus* and *A. camelliae* described by Kanmiya et al. [15], and simplified keys designated by Jansen and Porcelli [16] were employed to distinguish between *Camellia* and *Citrus* spiny whiteflies.

To confirm the morphological identification of mitochondrial DNA markers of *cytochrome c oxidase* I (COI-1) using the LCO1490/HCO2198 primer set [29], *C1-J-2195/L2-N-3014* (COI-2; [30]) and *cytochrome b* (COB) were used. Species-specific primers designed by Uesugi and Sato [23] were also applied to avoid misamplification due to the parasitism of parasitoid wasps. In addition, haplotype-specific primers were designed to confirm strain *A. camelliae* without sequencing based on the sequence data accession nos. LCO88497.1, AB786712.1, AB786713.1, and AB786714.1 (AC-55F: AGRAGTGAGTCTGGTAAGTTGG/ACB1-267R: ACCACCTAGAGTTGCCAACC). PCR conditions were set as follows: pre-denaturation at 95 °C for 2 min, continued with 35 cycles of denaturation at 98 °C for 10 s, annealing temperature 50 °C–52 °C for 30 s, and 72 °C for 1 min, with an extension period at 72 °C for 4 min.

### 2.4. Nested PCR for Determining Wolbachia Infections and MLST Sequencing

*Wolbachia* surface protein (*wsp*) typing was performed to detect *Wolbachia* infections using primer 81F/691R [31]. To confirm the negative results and obtain a fair sequence length of ~500 bp, nested PCR was also performed using primer *wspNesF/wspNesR* [32] to avoid false-negative results from PCR [32]. The monthly positivity rates of *Wolbachia* were monitored from March 2021 to February 2022. The monthly average temperature data were retrieved from Japan Meteorological Agency (https://www.data.jma.go.jp/; accessed on 31 March 2022) for Kikukawa–Makinohara (Shimada city, Shizuoka Prefecture). The associations between *Wolbachia* positivity rates and the average temperatures in the location sample (Shimada city) were estimated using the logistic regression analysis in the R software. Generalized linear models (GLMs; logit link and a binomial distribution) were constructed using the positivity rate as the response variable and the average temperature as an explanatory variable. The p-values for logistic regression were tested using the Wald test, with the level of significance set at *p* ≤ 0.05).

The single-gene profiling of the *16S rRNA* gene of *Wolbachia* was conducted for comparison using the *wspecF/wspecR* primer [33]. The diversity of *Wolbachia* was evaluated by profiling five housekeeping genes using a primer combination designed by [34] and using the *ftsZUniF/ftsZUniR* primer [33].

PCR was conducted in a total volume of 20 μL GoTaq^®^ Green Master Mix (1 μL DNA template, 1 μL of each primer, 7 μL of double-distilled H_2_O, and 10 μL of GoTaq). The PCR process used in this study included several steps, starting with pre-denaturation at 98 °C for 2 s, followed by 35 cycles at 98 °C for 10 s. It had an annealing temperature for 50 s, and 72 °C for 1 min, with a final extension period at 72 °C for 4 min. The PCR products were visualized via 1.5% agarose gel electrophoresis. The PCR products were direct-forward-sequenced after purification using ExoSAP-IT (Thermo Fisher Scientific Baltics UAB, Vilnius, Lithuania).

### 2.5. Bacteriophage Detection and Wolbachia Phenotypic Characteristic Determination

The bacteriophage of *Wolbachia* (phage WO) was detected by targeting the capsid protein gene *orf7* of phage WO, WO-F/R [35] and WO-SUF/R [36] comparison phage WO diversity. The genes related to the CI and feminization, such as ankyrin genes *pk1* and *pk2* [37,38] and non-ankyrin genes *cifA* and *cifB* [39,40], were targeted for the detection of a possible mechanism of speciation with the *Wolbachia* CI strain.

### 2.6. DNA Sequencing and Phylogenetic Analysis

The amplified fragments of representative samples were directly sequenced by a commercial Sanger sequencing service (Fasmac; Atsugi, Japan), and further analysis was conducted from the obtained sequences. Sequence similarity was analyzed using BLAST [41] on the nucleotide sequences deposited in the NCBI GenBank databases. Sequences were aligned with ClustalW using MEGA X [42]. Phylogenetic analyses were conducted using the maximum likelihood (ML) method [43], and 1000 bootstrap replicates were performed. Evolutionary analysis via the ML method (timetree) was generated using the RelTime method [44], calculated with the ML method, and the Tamura–Nei model [43] using MEGA X.

### 2.7. Genetic Differentiation, Network Analysis, and Recombination Test of Wolbachia

The net genetic divergence between and within groups (p-distance) of *wsp* and *16S rRNA* of *Wolbachia* was estimated using MEGA X [42]. The genetic parameters of the population, the number of segregating sites [45], the number of haplotypes (*h*), haplotype diversity (*Hd*) [46], and nucleotide diversity (π/bp) [46] were estimated using DNASP version 6 [47]. Using this software, a neutrality test was conducted, which examined population expansion by analyzing deviations from selective neutrality using Tajima’s D [48] and Fu and Li’s D* and F tests [49]. A median-joining 16S rRNA of the *Wolbachia* haplotype network was constructed using the Network 10 software [50]. The negative Tajima’s D and Fu and Li’s D* and F* values, according to Tseng et al. [51], may indicate a recent population expansion, purifying selection, or genetic hitchhiking, whereas positive values are more likely to indicate a population bottleneck, genetic structure, and/or balancing selection.

Putative recombinant strains in multiple sequence alignments from single-gene typing and MLST were analyzed using RDP5 [52]. Nine methods were employed in the analysis as follows: RDP [53], GENECONV [54], BootsScan [55], MaxChi [56], ChiMaera [57], SiScan [58], Phylpro [59], LARD [60], and 3Seq [61]. The default search parameters of the program were used. The acceptable *p*-value was <0.05

## 3. Results

### 3.1. Morphomolecular Identification

The molecular identification of *A. camelliae* cryptic species complex using universal primers targeting mitochondrial genes, such as COI and COB, was sensitive to the amplification of genes of parasitoid wasps rather than whiteflies. Parasitoid wasps belonging to the genera *Encarsia* and *Eretmocerus* were detected on most representative samples from the fields, such as A1V20, A1W20, B1V20, A1V20, F2X20, A1X21, and A2Z21 (Table 2). Only a few of them were closely related to the sequence data of whiteflies. Using COI-1 typing, *A. camelliae* haplotype B1 (A1W20-A7) was 99.7% identical to *A. spiniferus* (no. KJ437166.1), whereas *A. spiniferus* demonstrated 83.18% reference to *Aleurocanthus aracae* (no. MZ301225.1). Therefore, *Aleurocanthus* species-specific (TSW and OSW) primers [19,23] and haplotype-specific (AC55F/ACB1-267R) primers are useful to overcome this obstacle. 

**Figure 2:**
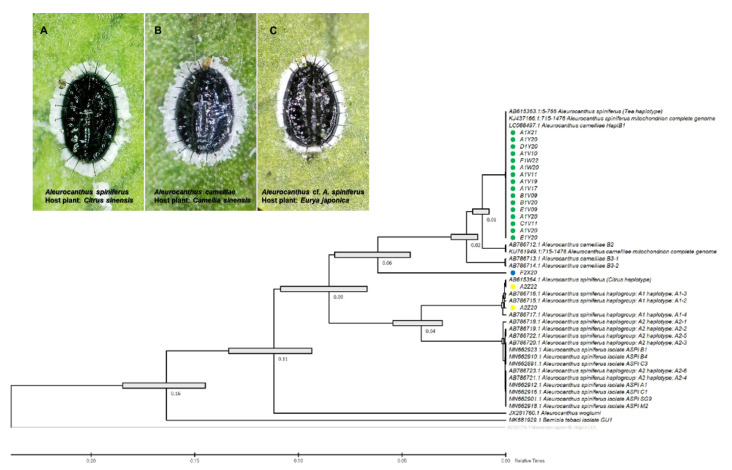
Evolutionary analysis via maximum likelihood method (timetree) based on the partial sequence mitochondrial COI (COI-2) gene of the *A. camelliae* cryptic species complex. Yellow circles are isolates of the *A. spiniferus* haplogroup A1 (**A**); green circle isolates are the *A. camelliae* haplotype B1 (**B**); and the blue circle is an isolate of the *Aleurocanthus* cf. *A. spiniferus* (**C**). Nodes with error bars were indicated in grey bars. *Nilaparvata lugens* (no. AB325705.1) were assigned as an outgroup. The evolutionary time was predicted by the relative time (Rt) scale bar.

The species-specific (TSW) and haplotype-specific primer (ACF55/ACB1267) primers were unable to confirm one isolate from *E. japonica* in Tokyo (F2X20) as *A. camelliae* haplotype B1. Despite the failure to amplify DNA using the TSW primer, the COI gene sequence obtained using the general primer tended to be grouped with *A. camelliae* (Figure 2). Thus, morphological confirmation was conducted, and it was found that isolate F2X20 was related to *A. spiniferus* instead of *A. camelliae*, with features such as a zig-zag arrangement of submedian abdominal spines and having more than 200 marginal teeth. Therefore, this isolate conformed to *A. spiniferus (Aleurocanthus* cf. *A. spiniferus).* The F2X20 isolate or *Aleurocanthus* cf. *A. spiniferus* sequence was identical to *Aleurocanthus* sp. (no. KY835557.1 and no. KY836994.1), with >81% similarity. Using COI-2, this isolate was referred to as *Tetraleurodes acaciae* (no. MT901108.1).

### 3.2. Positivity and Infection Rates of Wolbachia

**Figure 3:**
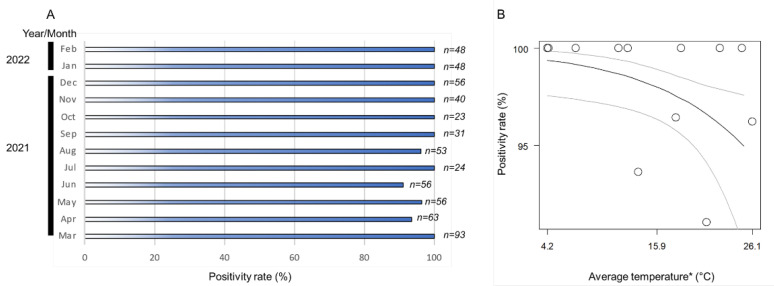
(**A**) Nested PCR detection of *the wsp* gene revealed a positivity rate range of 91–100%; (**B**) logistic regression analysis on fixed infection across the seasonal temperature. Black line indicates regression line, while grey lines are upper and lower thresholds of 95% confidence interval of predicted line. Regression coefficient was significant (Wald test; *p* < 0.05). (*) Monthly average temperature data were retrieved from the Japan Meteorological Agency (https://www.data.jma.go.jp/; accessed on 31 March 2022) for Kikukawa–Makinohara (Shimada city, Shizuoka Prefecture).

The monthly positivity rates (ratio of positive samples per assessed samples) of *Wolbachia* in the *A. camelliae* haplotype B1 ranged from 91% to 100% (Figure 3A). The positive rates remained high across the seasonal temperature, but as the temperature increased (>26 °C), the positive rates tended to decrease (Figure 3B). The high monthly positivity rate confirmed a high infection rate (overall samples assessed) detected in *A. camelliae* from *C. sinensis* (96.5%), while a medium rate was detected in *C. japonica* (40%), and a low rate was detected in *C. sasanqua* (6.7%) (Table 3). As only a single isolate was examined from *E. japonica*, it was difficult to estimate their actual infection rate. *A. spiniferus* is an uninfected population, as individuals were trans-parasitized by *Eretmocerus* under laboratory conditions, as strongly indicated by their identical strain, *Wolbachia*, despite some individual nymphs being positively infected (A2Z20-1; see Table 2). A similar case might have also occurred in *Aleurocanthus* cf. *A. spiniferus*. Only one individual (F2X20-3; see Table 3) was confirmed to be infected by *Wolbachia* and simultaneously parasitized by the parasitoid wasp.

### 3.3. Genetic Diversity of Wolbachia

**Figure 4:**
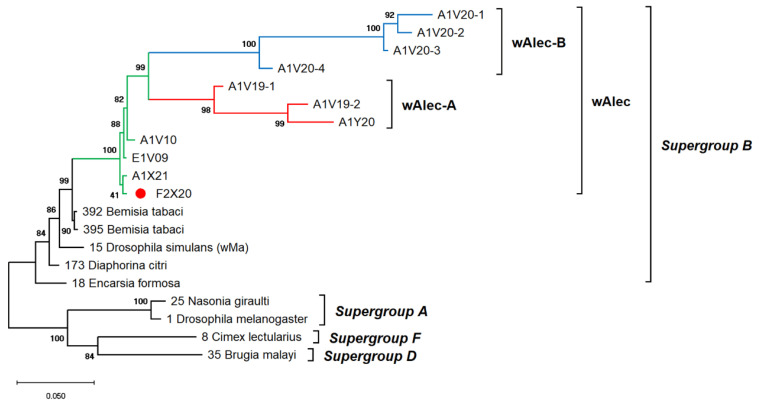
ML phylogenetic tree of *Wolbachia* MLST genes. The tree was constructed based on multiple alignments of concatenated DNA sequences encoding *gatB*, *coxA*, *hcpA*, *ftsZ*, and *fbpA* in ~2 kbp. Bootstrap values are shown for all nodes. A single lineage of *wAlec* (green line) evolved into two distinct branches of recombinant strains subgroups A (red) and B (blue). The *wAlec* also infected *Aleurocanthus* cf. *A. spiniferus* (red circle).

The genetic diversity of *Wolbachia* infects *A. camelliae* is difficult to estimate. Single-gene typing using *wsp* indicated an exceptionally low diversity of *Wolbachia*, which only consisted of three haplotypes (Hd: 0.1) and nucleotide diversity (π: 0.00099). Other genes, such as the *16S rRNA* of *Wolbachia,* detected among *A. camelliae* populations, were found to be extremely diverse (Hd: 0.8), with 21 haplotypes and diversity among nucleotides (π: 0.02292) (Table 4). Through MLST, *Aleurocanthus* spp., notably *A. camelliae* haplotype B1 and *Aleurocanthus* cf. *A. spiniferus*, seemed to harbor a single group of *Wolbachia*, namely, *wAlec*, as indicated by the monophyletic clade among these strains. The *wAlec* strains developed subgroups A and B (Figure 4). These strains were grouped into the *Wolbachia* supergroup B with other strains such as *wBtab*, *wMa*, *wDcit*, and *wEfor*. 

### 3.4. Phage WO Detection and Wolbachia Phenotypic Screening

The low genetic distance or sequence dissimilarity (<1%) of phage WO-infected *Wolbachia* in *A. camelliae* from *C. sinensis* (A1V20) and *E. japonica* (A1X21), along with *Aleurocanthus* sp. in *E. japonica* (F2X20), indicated that they harbored a single strain of phage WO, namely, *WOAlec* (Table 5). This was also confirmed by the sequences obtained using the new primer set of WOSUF/R. The genes that regulated CI phenotypes in *Wolbachia* from ankyrin and non-ankyrin genes were not detected in the phage WO strain.

### 3.5. Recombination and Haplotype Diversity of Wolbachia

**Figure 5:**
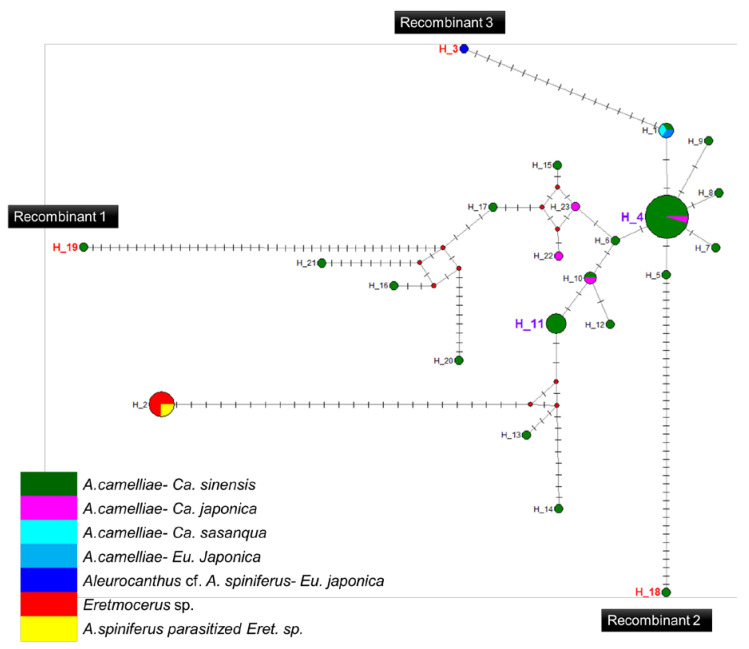
Haplotype network diagram inferred from the *16S rRNA* gene of *Wolbachia*. Red nodes are median vectors. Striped lines indicate the number of nucleotide mutations.

A high prevalence of putative recombinant strains was consistently detected using the GENECONV, ChiMaera, and Phylpro tests. Ten strains were identified in single-gene and MLST-aligned sequences. Other tests, such as RDP, BootScan, ChiMaera, and SiScan, confirmed four to nine recombination events (Table 6). Recombination was observed in the *wsp* of E3V20 or *Pealius euryae* in *C. sinensis* from Kyoto with the main parent, *A. camelliae*, from the same host and location (E1V20; Table 6). In addition, haplotype 18 (A1V20-27), haplotype 19 (A1V20-10), and haplotype 3 (F2X20) experienced recombination on their 16S rRNA of the *Wolbachia* gene (Figure 5; Table 6). Based on the MLST sequences, the major parent of recombinant strains of *wAlec* subgroup B (A1V20-3, A1V20-2, A1V20-4, and A1V20-1) was the strain from *wAlec* subgroup A (A1V19-2 and A1V19-1) with similarity of 93.1–96.4% (Figure 4; Table 6). In addition, *wAlec* subgroup A (A1V19-2 and A1V19-1) seemed to have A1Y20 from the same subgroup as their major parent (Figure 5; Table 6).

## 4. Discussion

Whiteflies are sap-sucking insects belonging to the family Aleyrodidae, which consists of >1550 species, mostly belonging to the subfamilies Aleurodicinae and Aleyrodinae [62]. The morphological identification of whiteflies (Hemiptera: Aleyrodidae), which focused on the characteristics of puparium, has been suggested to be limited and might not even be genus-specific [63] to the *Aleurocanthus* genus [16]. The current morphological characteristics, number of submarginal spines, number of marginal teeth, arrangement of submedian abdominal spines, and microscopic papillae failed to separate *Aleurocanthus* cf. *A. spiniferus* (F2X20) from *A. spiniferus*, which is genetically different from *A. spiniferus* and *A. camelliae* (Figure 2). This confirms the existence of the novel cryptic species complex of *A. camelliae* in Japan.

**Figure 6:**
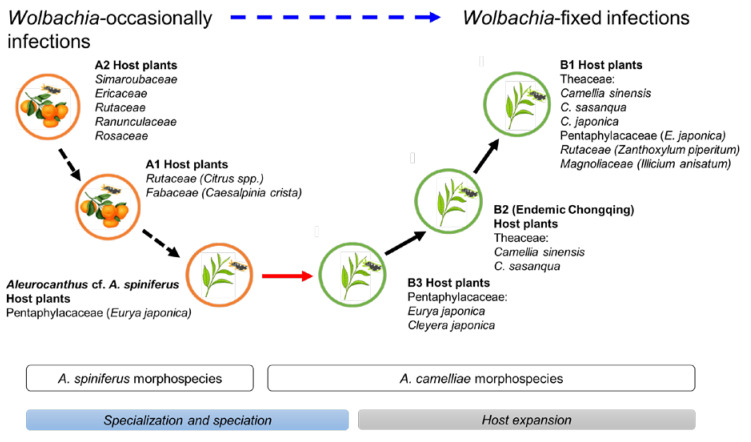
Hypothetical diagram of the evolutionary history of *A. camelliae* cryptic species. Predicted speciation time (see Figure 2) among *A. spiniferus* morphospecies occurred at the relatively same time (Rt 0.06) and was significantly separated from the predicted speciation time of *A. camelliae* morphospecies (Rt 0.01–0.02). The oscillation in the host plant range represents specialization (black-dashed arrow) and speciation (red arrow; blue bar) to host expansion (black arrow; gray bar). The hierarchical infection status of *Wolbachia* might be associated with the morphospecies (blue-dashed arrow).

The *Aleurocanthus* genus consists of at least 78 recorded species, and most species are specific to one or two families of host plants [64]. Among those species, *A. woglumi* and *A. spiniferus* are well-known as extremely polyphagous whiteflies that are widely distributed worldwide. *A. woglumi* inhabits more than 37 host plants, while *A. spiniferus* inhabits more than 19 families of host plants [64]. The oscillation hypothesis suggests a link between the host plant and geographical range as a contributing factor in increasing diversification rates [65], indicating that the occurrence of diversity in phytophagous insects may be promoted through oscillation in the host plant range. We believe that the current findings also support this hypothesis (Figure 6). The discovery of the novel cryptic species, *Aleurocanthus* cf. *A. spiniferus*, linked the history of adaptation among *A. spiniferus* and *A. camelliae*, suggesting that the most recent common ancestor of *A. camelliae* morphospecies is the *A. spiniferus* morphospecies that inhabits *Theaceae* sensu lato (*Pentaphylacaceae*). The cladogenesis of *Aleurocanthus* cf. *A. spiniferus* tended to lean toward *A. camelliae* instead of *A. spiniferus* (Figure 2), perhaps correlating to the host plants’ group. Theaceae and Pentaphylacaceae are plant families that belong to the same order of *Ericales* [66]. Therefore, further research on the oscillation hypothesis for the cryptic speciation of *A. camelliae* may benefit from investigations of how *A. spiniferus* inhabits another plant of the order *Ericales* such as *Diospyros khaki* (Ebenaceae) in Japan [67].

In the cryptic species complex of *A. camelliae*, a different pattern of *Wolbachia* infection was found (Table 3). Whiteflies morphologically identical to *A. spiniferus* (*Aleurocanthus* sp. in *E. japonica* and *A. spiniferus* in *Citrus*) were grouped into uninfected populations, whereas *A. camelliae* B1 from *C. sinensis* was considered the *Wolbachia*-infected population. *Wolbachia* infections have been known to significantly affect the structure and mitochondrial diversity of host insects [10,68], leading to cryptic speciation [3]. A similar case has recently been reported in the *Wiebesia pumilae* cryptic species (Hymenoptera: Agaonidae), which produce hierarchical *Wolbachia* infection patterns [69]. The spread barrier produced by cryptic species or a different ancestor host population containing *Wolbachia* CI strains may be the reason for the distinct infection status among cryptic species. However, *wAlec* is not a *Wolbachia* CI strain (Table 5), but it does not rule out the possibility that *wAlec* had a role in speciation since the retention of *Wolbachia* CI strains for long-term prognosis following secondary contact and spatial reunification of two allopatrically separated populations of a species is normally not favorable. The *wAlec* CI strains may exist and could have aided the emergence of further reproductive isolation through the process of reinforcement [70] and maintained population differentiation [71]. 

The intraspecies or intrapopulation infection rates might also vary following the host preferences of *A. camelliae* itself. Lower infection rates were found in *A. camelliae*-B1-infesting alternative hosts, such as *C. japonica* and *C. sasanqua*. *Wolbachia* titer is not only maternally inherited, but it can also be horizontally transmitted [71] or eventually lost [72]. Fixed infection in *A. camelliae* haplotype B1 inhibited *C. sinensis* (Figure 3A), suggesting that *wAlec* might have nutritional mutualism such as synthesizing biotin, which might explain the transition from facultative symbiosis to obligate mutualism [73]. 

This study also provided novel evidence of the recombination event of *Wolbachia* in the whitefly community in *C. sinensis*. *Wolbachia*-strain-infected *P. euryae* (E3V20) was derived from *Wolbachia*-infected *A. camelliae* (E1V20). Both were collected from Kyoto. The recombination was also observed in the population of *A. camelliae* that were infected by the *wAlec* group strains. Notably, *wAlec* subgroup B (A1V20-3, A1V20-2, A1V20-4, and A1V20-1) was derived from *wAlec* subgroup A (A1V19-2 and A1V19-1) as major parents, and the samples were collected in 2020 and 2019 from the same location, respectively. The recombination is likely to be essential for *Wolbachia* adaptation to escape Muller’s ratchet, a process leading to the accumulation of mildly deleterious alleles, which is a problem for symbionts that face a population bottleneck in each generation [74,75]. Production of new recombinants results in *Wolbachia* strains with fewer harmful mutations and greater genetic variety, allowing them to use a wider range of hosts. This phenomenon is also well-known in pathogenic bacteria [76,77,78]. High recombination rates might also indicate a high incidence of horizontal transmission. Bacterial symbionts often maintain intermediate symbiont genome sizes and substantial functional genetic variation through horizontal transmission and recombination [79]. Further analysis is required to determine whether the mechanism of high recombination in *wAlec* results in the loss of CI strains. The bioassay confirmation of the CI phenotype of *wAlec* and/or trans-infection of *Wolbachia* CI strains, e.g., *wMel* [80], might be useful as a biological control method to contain the *A. camelliae* cryptic species complex [80]. 

The detection of positive infection in some parasitized nymphs of the *A. spiniferus* morphospecies and *Eretmocerus* sp. (Table 2 and Table 3) revealed the possibility of parasitoids as vectors of *Wolbachia* [81,82] or the reverse transmission pathway from hosts to parasitoids [83]. *Eretmocerus* sp. parasitizing *A. camelliae* is a newly recorded occurrence in Japan. Historically, *Encarsia smithi* is the only parasitoid wasp of the black spiny whitefly species (*A. camelliae* and *A. spiniferus*) in Japan [19,84,85,86,87]. Thus, further studies are needed to identify the *Eretmocerus* species parasitizing *A. camelliae* and their origin in order to provide comprehensive information regarding the potential natural enemies of *A. camelliae*.

## Figures and Tables

**Table 1:** Whitefly collection.

Whitefly Species	Haplotype	Locality (Prefecture)	Host Plant	Year	Label Isolate *
*A. camelliae*	B1	Shizuoka	*C. sinensis*	2010	A1V10
	B1	Shizuoka	*C. sinensis*	2011	A1V11
	B1	Shizuoka	*C. sinensis*	2017	A1V17
	B1	Shizuoka	*C. sinensis*	2018	A1V18
	B1	Shizuoka	*C. sinensis*	2019	A1V19
	B1	Shizuoka	*C. sinensis*	2020	A1V20
	B1	Shizuoka	*C. japonica*	2020	A1W20
	B1	Shizuoka	*E. japonica*	2021	A1X21
	B1	Shizuoka	*C. sasanqua*	2020	A1Y20
	B1	Shiga	*C. sinensis*	2009	B1V09
	B1	Shiga	*C. sinensis*	2020	B1V20
	B1	Mie	*C. sinensis*	2011	C1V11
	B1	Osaka	*C. sasanqua*	2020	D1Y20
	B1	Kyoto	*C. sinensis*	2009	E1V09
	B1	Kyoto	*C. sinensis*	2020	E1V20
	B1	Tokyo	*E.japonica*	2022	F1W22
*A. spiniferus*	A1	Shizuoka	*Ci.* *sinensis*	2020	A2Z20 ^a^
	A1	Shizuoka	*Ci.* *sinensis*	2021	A2Z21
	A1	Shizuoka	*Ci.* *sinensis*	2022	A2Z22
	?	Tokyo	*E. japonica*	2020	F2X20
*P. euryae*		Shizuoka	*E. japonica*	2021	A3X21
		Shizuoka	*C. sinensis*	2020	A3V20
		Kyoto	*C. sinensis*	2020	E3V20

(*) Labeling order: prefecture, whitefly species, host plant, and year. In data analysis, some isolates were added label (-No.), which represented the individual sample number analyzed. ^a^ Colony reared on the citrus leaves in a cage (34 × 34 × 34 cm) under laboratory conditions (23 °C; 16:8 h light/dark photoperiod) for breeding parasitoid wasps.

**Table 2:** Identification of mitochondrial genes using BLAST and the *Wolbachia* infection status.

Gene	Isolates	Type ^a^	Close Relative	% Similarity	Source	Infection
*COI-1*	A1V20-1	B1	*Encarsia* sp.	90.94	KJ444561.1	(+)
	A1W20-1	B1	*Encarsia inquirenda*	92.74	MH928989.1	(+)
	A1W20-2	B1	*Encarsia perniciosi*	90.28	JQ083717.1	(+)
	A1W20-3	B1	*A. spiniferus*	99.38	KJ437166.1	(+)
	D1Y20	B1	*A. spiniferus*	99.53	KJ437166.1	(+)
	B1V20	B1	*Encarsia obtusiclava*	90.17	MG813798.1	(−)
	F2X20-1	-	*Aleurocanthus* sp.	81.75	KY835557.1	(−)
	F2X20-2	-	*Aleurocanthus* sp.	81.95	KY836994.1	(−)
	A1V20-2	B1	*Eretmocerus orchamoplati*	88.78	JF750712.1	(+)
	A2Z20-1 ^b^	-	*E. orchamoplati*	84.62	JF750714.1	(+)
	A2Z20-2 ^b^	-	*Aleurocanthus arecae*	83.18	MZ301225.1	(−)
*COI-2*	F2X20-4	-	*E. smithi* type 2	99.46	AB786724.1	(−)
	F2X20-3	-	*E. smithi* type 1	97.82	AB786726.1	(+)
	F2X20-5	-	*T. acaciae*	80.72	MT901108.1	(−)
	A2Z21-1	-	*E. smithi* type 1	99.32	AB786726.1	(−)
	A2Z21-2	-	*E. smithi* type 1	99.32	AB786726.1	(−)
	A1V20-3	B1	*E. smithi* type 1	99.57	AB786726.1	(+)
	A1V20-4	B1	*E. smithi* type 1	98.29	AB786726.1	(+)
*COB*	F2X20-3	-	*Encarsia formosa*	86.44	MG813797.1	(+)
	F2X20-4	-	*E. formosa*	86.49	MG813797.1	(−)
	A1V20-5	B1	*E. formosa*	86.39	MG813797.1	(+)
	A1V20-6	B1	*Eretmocerus* sp.	84.89	KX714964.1	(+)
	A1V20-7	B1	*E. formosa*	85.91	MG813797.1	(+)
	A1X21	B1	*Eretmocerus* sp.	85.16	KX714964.1	(+)
	A2Z21-3	-	*E. formosa*	86.57	MG813797.1	(−)
	A2Z21-4	-	*E. formosa*	86.26	MG813797.1	(−)

^a^ The confirmation type is based on haplotype-specific amplification. ^b^ Laboratory reared.

**Table 3:** Infection status of *Wolbachia* using nested PCR.

Species	Host	No. Samples Assessed	mtCOI Gene of Host Amplification	Positive Infection (Nested PCR)	Infection Rate ^c^ (%)
*A. camelliae*	*C. sinensis*	738	728	703	96.5
	*C. sasanqua*	30	30	2	6.7
	*C. japonica*	15	15	6	40
	*E. japonica*	1	1	1	100 ^a^
*A. spiniferus*	*C.* *sinensis*	104	103	2	1.9
*Aleurocanthus* cf. *A. spiniferus*	*E. japonica*	40	40	1	2.5
*E. smithi*	*A. spiniferus*	16	16	0	0
*Eretmocerus*	*A. camelliae*	7	7	7	100
	*A. spiniferus* ^b^	1	1	1	100
Total		952	941	722	

^a^ Not the actual infection rate due to the limited sample. ^b^ Laboratory reared. ^c^ Proportion of positive infection and mtCOI host amplification.

**Table 4:** Haplotype diversity of *Wolbachia* in *A. camelliae* haplotype B1 was estimated from a 364 bp *wsp* and 385 bp 16S rRNA of *Wolbachia* gene fragments.

Gene	Sample Pool	N	S	h	Molecular Diversity Indices			Neutrality Tests	
					Hd	π	k	Tajima’s D (*P*)	Fu and Li’s F (*P*)
*wsp*	*A. camelliae* populations	30	5	3	0.1	0.00099	0.33	−2.00763 (<0.05) *	−3.34142 (<0.02) **
	Associated populations *	8	122	8	1.0	0.13692	46.14	−0.60085 (>0.10) ^ns^	−0.61175 (>0.10) ^ns^
*16S rRNA*	*A. camelliae* populations	51	85	21	0.8	0.02292	7.71	−2.31567 (<0.01) **	−3.93027 (<0.02) **
	Associated populations *	9	36	2	0.2	0.02026	7.33	−1.99788 (<001) **	−2.48500 (<0.02) **

N, number of sequences; S, number of segregating or polymorphic sites; h, number of haplotypes; Hd, haplotype diversity; π, nucleotide diversity; k, mean number of nucleotide differences. * Associated populations are *Wolbachia* sequence collected from the other whiteflies and parasitoid wasps surrounding *A. camelliae*. ^ns^ *p* > 0.10, * *p* < 0.05, and ** *p* < 0.02, level of significance of Tajima’s D and Fu * Li’s F tests.

**Table 5:** Diversity of phage WO (*WOAlec*) and phenotypic screening.

Isolates		Sequence Dissimilarity				Phenotypic Screening					
		1	2	3	4	*pk1a*	*pk1b*	*pk2b1*	*pk2b2*	*cifA*	*cifB*
1	A1V20-1		0.000	0.004	0.004	(−)	(−)	(−)	(−)	(−)	(−)
2	A1V20-2	0.000		0.004	0.004	(−)	(−)	(−)	(−)	(−)	(−)
3	F2X20	0.006	0.006		0.004	(−)	(−)	(−)	(−)	(−)	(−)
4	A1X21	0.006	0.006	0.006		(−)	(−)	(−)	(−)	(−)	(−)

**Table 6:** Intragenic recombination in *wAlec* by using nine different methods implemented in RDP5 software.

Gene	No. Events ^a^	Putative Recombination ^b^	Major Parent ^c^ (% Similarity)	Minor Parent ^d^ (% Similarity)	Analysis									GENECONV	
					R	G	B	M	C	S	P	L	3S	Start	End
*wsp*	1	E3V20	E1V20 (82.6)	Unknown	(−)	+	(−)	+	+	(−)	+	(−)	(−)	94	254
*16S rRNA*	2	A1V20-10	A1V20-13 (96.3)	Unknown	(−)	+	(−)	+	+	+	+	(−)	(−)	284	372
	3	A1V20-27	A1V20-13 (96.7)	Unknown	+	+	(−)	(−)	+	(−)	+	(−)	(−)	237	376
	4	F2X20	A1V20-13 (95.2)	Unknown	+	+	(−)	+	+	(−)	+	(−)	(−)	288	380
MLST	5	A1V20-3	A1V19-1 (94)	Unknown	+	+	+	+	+	(−)	+	(−)	(−)	409	755
	6	A1V20-2	A1V19-1 (94.2)	Unknown	+	+	+	+	+	(−)	+	(−)	(−)	396	764
	7	A1V20-4	A1V19-2 (93.1)	*Drosophila simulans* (89.4)	+	+	+	+	+	+	+	(−)	(−)	1	362
	8	A1V20-1	A1V19-2 (96.5)	*D. simulans* (97)	+	+	+	+	+	+	+	(−)	(−)	131	361
	9	A1V19-2	A1Y20 (94.7)	A1V20-4 (99.8)	+	+	+	+	+	+	+	(−)	(−)	1250	∞~
	10	A1V19-1	A1Y20 (95.2)	*Brugia malayi* (92.8)	+	+	(−)	+	+	(−)	+	(−)	(−)	∞~	628

^a^ Recombination events detected by more than two analysis methods. ^b^ Putative recombinant: strains experienced recombination. ^c^ Major parent: parent contributing the larger fraction of the putative recombinant sequence. ^d^ Minor parent: parent contributing the smaller fraction of the putative recombinant sequence R, RDP; G, GENECONV; B, BootScan; M, MaxChi; C, ChiMaera; S, SiScan; P, Phylpro; L, LARD; 3S, 3Seq. ∞~: undetermined.

## Data Availability

Publicly available datasets were analyzed in this study.
